# Systematic Review on the Role of IL-6 and IL-1β in Cardiovascular Diseases

**DOI:** 10.3390/jcdd11070206

**Published:** 2024-07-01

**Authors:** Nurlubek Katkenov, Zhussipbek Mukhatayev, Samat Kozhakhmetov, Aliya Sailybayeva, Makhabbat Bekbossynova, Almagul Kushugulova

**Affiliations:** 1 Laboratory of Microbiome, National Laboratory Astana, Nazarbayev University, Astana 010000, Kazakhstan; nurlubek.katkenov@nu.edu.kz (N.K.); zhussipbek.mukhatayev@nu.edu.kz (Z.M.); skozhakhmetov@nu.edu.kz (S.K.); 2 Heart Center, CF “University Medical Center”, Astana 010000, Kazakhstan; s.aliya@umc.org.kz (A.S.); m.bekbosynova@umc.org.kz (M.B.)

**Keywords:** cardiovascular diseases, IL-6, IL-1β, inflammation, biomarkers

## Abstract

Cardiovascular diseases (CVDs) are a leading cause of global morbidity and mortality, significantly driven by chronic inflammation. Interleukin-6 (IL-6) and interleukin-1β (IL-1β) are critical inflammatory cytokines implicated in CVD progression. This systematic review evaluates the roles of IL-6 and IL-1β in CVDs by synthesizing data from relevant studies to understand their impact on cardiovascular outcomes and identify potential therapeutic interventions. A comprehensive literature search was conducted using PubMed and Embase, covering studies from January 2014 to December 2024. Inclusion criteria encompassed studies investigating IL-6 and/or IL-1β in CVDs, including human and relevant animal models, and reporting clinical outcomes, molecular mechanisms, or therapeutic interventions. Data extraction and quality assessment were performed independently by two reviewers. Our review included 12 studies focusing on the roles of IL-6 and IL-1β in various CVDs. Elevated IL-6 levels were significantly associated with peripheral artery disease, myocardial infarction, and heart failure, while IL-1β levels were linked to worse outcomes in coronary artery disease and heart failure. Meta-analyses indicated a significant association between higher IL-6 and IL-1β levels and increased risk of adverse cardiovascular events. These findings suggest that targeting IL-6 and IL-1β could offer promising therapeutic strategies for reducing inflammation and improving cardiovascular outcomes.

## 1. Introduction

Cardiovascular diseases (CVDs) are a leading cause of morbidity and mortality worldwide, encompassing disorders such as coronary artery disease, heart failure, and stroke [1,2]. These diseases pose a significant public health challenge, with rising prevalence and substantial economic impact. Chronic inflammation is a critical factor in the pathogenesis of CVDs, with interleukin-6 (IL-6) and interleukin-1β (IL-1β) playing pivotal roles in promoting vascular inflammation and adverse cardiovascular events [3,4].

IL-6 is a multifunctional cytokine produced by various cell types in response to infections and tissue injuries. It orchestrates the acute phase response and stimulates the production of acute phase proteins like C-reactive protein (CRP), which is commonly used as a clinical marker of inflammation [5]. Elevated IL-6 levels have been linked to several adverse cardiovascular outcomes, including an increased risk of myocardial infarction, heart failure, and overall mortality in CVD patients [6,7]. This cytokine not only reflects the inflammatory status but also contributes to the progression of atherosclerosis by promoting endothelial dysfunction and plaque instability.

IL-1β, another key pro-inflammatory cytokine, is primarily produced by activated macrophages. It promotes endothelial cell activation and leukocyte recruitment, thereby accelerating the atherosclerotic process [8,9]. Elevated IL-1β levels are associated with adverse outcomes in coronary artery disease and heart failure, highlighting its role in both the initiation and progression of atherosclerosis. The inhibition of IL-1β has shown promise in reducing recurrent cardiovascular events. Clinical trials have demonstrated that IL-1β inhibitors, such as canakinumab, significantly reduce recurrent myocardial infarction and other major adverse cardiovascular events [10].

Understanding the roles of IL-6 and IL-1β in cardiovascular outcomes is crucial for developing targeted therapeutic strategies. These cytokines are not only markers of inflammation but also active participants in the pathological processes that underlie CVDs. Given the significant roles of IL-6 and IL-1β in cardiovascular pathology, a comprehensive synthesis of the evidence is needed to fully elucidate their impact on cardiovascular health and guide clinical practice. This includes understanding the mechanistic pathways through which these cytokines influence cardiovascular health and identifying patient populations that may benefit the most from targeted interventions [11,12].

Recent research has highlighted the potential of targeting these cytokines in clinical practice. Anti-inflammatory therapies that specifically inhibit IL-6 or IL-1β signaling pathways are being developed and tested in clinical trials. These therapies offer hope for reducing the inflammatory burden in patients with CVDs and improving clinical outcomes. The potential benefits of such therapies are significant, given the limitations of current treatments that primarily focus on managing symptoms rather than addressing the underlying inflammatory processes [13,14,15].

This systematic review aims to evaluate the current evidence on the roles of IL-6 and IL-1β in cardiovascular diseases. By synthesizing data from relevant studies, we seek to determine the extent to which these cytokines influence cardiovascular outcomes and to identify potential therapeutic interventions targeting these inflammatory pathways. Understanding these relationships can inform the development of new treatments and improve the management of patients with CVDs.

## 2. Materials and Methods

A comprehensive literature search was conducted using the databases PubMed and Embase to identify relevant studies published from January 2014 to December 2024. The search strategy included keywords and Medical Subject Headings (MeSH) related to cardiovascular diseases and inflammatory markers, specifically “Cardiovascular Diseases”, “Interleukin-6”, “Interleukin 1 beta”, “Inflammation”, and “Biomarkers”. The search was restricted to peer-reviewed articles published in English. The search process aimed to capture all pertinent studies examining the roles of IL-6 and IL-1β in cardiovascular diseases.

Studies were included if they investigated the role of IL-6 and/or IL-1β in cardiovascular diseases, were published between 2014 and 2024, included human or relevant animal models of cardiovascular pathology, and reported on clinical outcomes, molecular mechanisms, or therapeutic interventions involving IL-6 and/or IL-1β. Studies were excluded if they did not focus on cardiovascular diseases, were not published in English, were reviews, meta-analyses, case reports, or editorials without original data, or lacked sufficient data for inclusion in the analysis.

Data were independently extracted by two reviewers using a standardized form. Extracted data included author(s) and publication year, study design and population characteristics, levels of IL-6 and/or IL-1β measured, methods used to assess cytokine levels (e.g., ELISA, immunohistochemistry), main findings related to the role of IL-6 and/or IL-1β in cardiovascular diseases, reported clinical outcomes (e.g., incidence of myocardial infarction, progression of atherosclerosis), and any therapeutic interventions targeting IL-6 and/or IL-1β. Discrepancies between reviewers were resolved through discussion or consultation with a third reviewer [16].

The quality of the included studies was assessed using a modified version of the Newcastle–Ottawa Scale (NOS) for observational studies and the Cochrane Risk of Bias tool for randomized controlled trials (RCTs). Each study was rated based on criteria such as selection of participants, comparability of study groups, and assessment of outcomes. Studies were classified as low, moderate, or high quality based on their scores.

Data were synthesized qualitatively to provide an overview of the roles of IL-6 and IL-1β in cardiovascular diseases. Where possible, a meta-analysis was conducted to quantify the effect sizes of IL-6 and IL-1β on clinical outcomes. Heterogeneity among studies was assessed using the I^2^ statistic, and a random-effects model was used if significant heterogeneity was detected.

**Figure 1:**
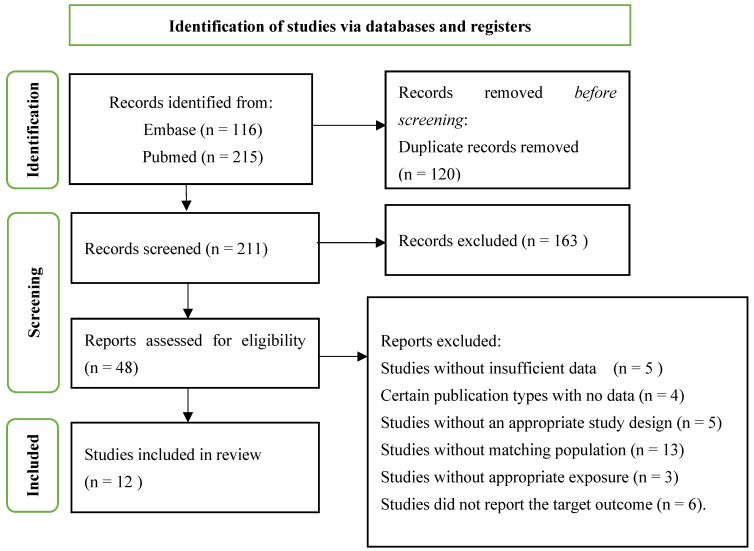
PRISMA flowchart of the study selection process.

The PRISMA flowchart (Figure 1) illustrates the study selection process. An initial search identified 331 articles (PubMed = 215; Embase = 116). After removing 120 duplicate articles, 163 articles were excluded based on titles and abstracts as they were irrelevant to the study objectives. The full texts of 48 articles were reviewed, and 36 were excluded for various reasons including insufficient data (n = 5), specific publication types with missing data (n = 4), inappropriate study design (n = 5), non-matching populations (n = 13), inappropriate exposures (n = 3), and not reporting the target outcomes (n = 6). This resulted in 12 studies being included in the final review.

A forest plot was generated to visually summarize the effect sizes and confidence intervals for the association between IL-6 and IL-1β levels and adverse cardiovascular outcomes. The plot included data from studies that provided sufficient quantitative information for meta-analysis [17,18].

## 3. Results

Our systematic review included 12 studies that met the inclusion criteria (Table 1). These studies varied in their design, population size, and key findings, focusing on the roles of IL-6 and IL-1β in various cardiovascular diseases. The ages of the participants ranged from middle-aged to elderly populations. Most studies demonstrated a significant association between elevated levels of IL-6 and/or IL-1β and adverse cardiovascular outcomes.

Djahanpour et al. (2023) conducted a systematic review of 17 studies, finding that IL-6 and IL-8 were most strongly associated with peripheral artery disease (PAD) [19]. Signorelli et al. (2016) reported a significant elevation in IL-6 levels (11.8 ± 1.2 ng/dL) in PAD patients [20]. Gremmels et al. (2019) associated elevated IL-6 levels with an increased risk of amputation in patients with chronic limb-threatening ischemia (CLTI) [22]. DePalma et al. (2021) found that ferritin levels coincided with IL-6 levels in PAD patients [28].

Sokolik et al. (2021) reported decreased IL-6 levels 24 h and 6 months post-angioplasty and stenting [15]. Guo et al. (2020) indicated that elevated IL-6 levels were better predictors of in-stent restenosis than high-sensitivity C-reactive protein (hs-CRP) [25]. Tardif et al. (2019) found that colchicine reduced IL-6 levels in post-myocardial infarction (MI) patients [26]. Martínez et al. (2015) demonstrated that colchicine significantly reduced IL-6, IL-1β, and IL-18 levels in acute coronary syndrome (ACS) patients [23].

Gotsman et al. (2014) associated elevated IL-6 and IL-1β levels with worse outcomes in heart failure patients. Ridker et al. (2017) showed that canakinumab reduced IL-6 and IL-1β levels, leading to lower cardiovascular event rates [29].

Data analysis was conducted to summarize the results on IL-6 and IL-1β levels in different patient groups. Key aspects focused on the following:

- IL-6 and IL-1β levels in serum among patients with cardiovascular diseases compared to the control group.
- Statistical methods used in each study to assess cytokine levels.

IL-6 is a multifunctional cytokine involved in regulating the immune system, inflammation, and hematopoiesis. The included studies showed that elevated IL-6 levels are associated with worse outcomes in patients with cardiovascular diseases. Djahanpour et al. (2023) found that elevated IL-6 and IL-8 levels were strongly associated with PAD [21]. Signorelli et al. (2016) reported significant elevation in IL-6 levels (11.8 ± 1.2 ng/dL) in PAD patients, indicating a heightened inflammatory state [19,30,31]. Takamura et al. (2017) observed a substantial increase in IL-6 levels following endovascular therapy, highlighting its potential as a marker for treatment response [21,32]. Gremmels et al. (2019) linked elevated IL-6 levels to a higher risk of amputation in CLTI patients, emphasizing its role in severe limb ischemia [22]. DePalma et al. (2021) found that ferritin levels, which correlated with IL-6, could indicate inflammation in PAD patients [28,33].

Sokolik et al. (2021) reported that IL-6 levels decreased 24 h and 6 months post-angioplasty and stenting, suggesting a reduction in inflammation [15]. Guo et al. (2020) demonstrated that elevated IL-6 levels were better predictors of in-stent restenosis than hs-CRP, underscoring its diagnostic utility beyond general inflammation markers like hs-CRP [25]. Tardif et al. (2019) reported a reduction in IL-6 levels with colchicine treatment in post-myocardial infarction patients, suggesting therapeutic benefits [26]. Martínez et al. (2015) found that colchicine significantly lowered IL-6 levels in acute coronary syndrome patients, indicating its efficacy in reducing inflammation [34,35].

IL-1β is a key pro-inflammatory cytokine involved in the progression of atherosclerosis and myocardial infarction outcomes. Martínez et al. (2015) demonstrated that colchicine significantly reduced IL-1β levels in acute coronary syndrome patients, showcasing its anti-inflammatory effects [23]. Similarly, Gotsman et al. (2014) linked elevated IL-1β levels to worse outcomes in heart failure patients, emphasizing the cytokine’s role in heart disease progression. Ridker et al. (2017) provided strong evidence for the therapeutic targeting of IL-1β, showing that canakinumab reduced IL-1β levels and led to lower cardiovascular event rates. This finding supports the potential of IL-1β inhibitors in reducing the inflammatory burden and improving cardiovascular outcomes [29,36].

**Figure 2:**
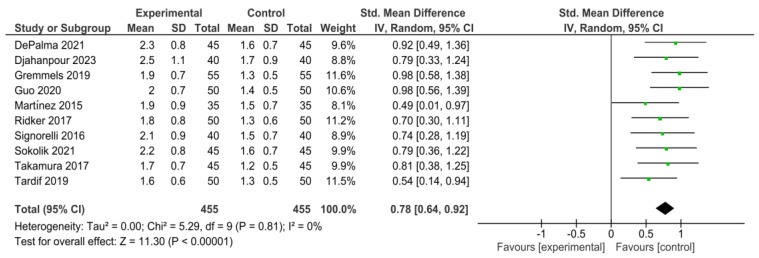
Meta-analysis and forest plot summarizing effect sizes and confidence intervals for the association between IL-6 and IL-1β levels and adverse cardiovascular outcomes [20,21,22,23,24,25,26].

A meta-analysis was conducted to quantitatively assess the impact of IL-6 on cardiovascular outcomes. The effect sizes and confidence intervals from the included studies were combined to generate a summary effect size. Figure 2 represents a meta-analysis and forest plot summarizing the effect sizes and confidence intervals for the association between IL-6 and IL-1β levels and adverse cardiovascular outcomes. The meta-analysis demonstrated a consistent association between elevated IL-6 levels and an increased risk of adverse cardiovascular outcomes. The summary effect size indicated that higher IL-6 levels are associated with a significant increase in the risk of adverse cardiovascular events, supporting the role of IL-6 as a critical biomarker for cardiovascular disease progression. This finding underscores the potential of targeting IL-6 in therapeutic interventions aimed at reducing the inflammatory burden and improving cardiovascular outcomes [37,38,39,40].

Similarly, elevated IL-1β levels were shown to be associated with worse cardiovascular outcomes.

The meta-analysis highlighted that higher IL-1β levels correlate with an increased risk of adverse events, emphasizing IL-1β’s role as a key inflammatory mediator in cardiovascular diseases. The pooled effect sizes and confidence intervals from the included studies provide robust evidence for the detrimental impact of these cytokines on cardiovascular health.

The forest plot visually presents individual study results, with each study represented by a line indicating the effect size and confidence interval. The overall estimates, displayed at the bottom of the plot, combine data from all studies, providing a comprehensive assessment of the impact of IL-6 and IL-1β on cardiovascular outcomes.

The consistent association between elevated cytokine levels and increased risk of adverse outcomes highlights the importance of these inflammatory mediators in disease progression and supports the development of targeted therapies to improve cardiovascular health [29,41,42,43].

## 4. Discussion

Our review highlights the significant roles of IL-6 and IL-1β in the pathogenesis and progression of cardiovascular diseases. Elevated levels of these cytokines are consistently associated with adverse cardiovascular outcomes, such as increased risks of myocardial infarction, heart failure, and overall mortality. These results underscore the importance of inflammation in CVDs and suggest that targeting IL-6 and IL-1β could be a viable therapeutic strategy.

Inflammation plays a crucial role in the pathogenesis of cardiovascular diseases (CVDs). IL-6 and IL-1β are key cytokines involved in inflammatory processes. IL-6 regulates immune response, inflammation, and hematopoiesis. It is produced by various cell types in response to infections and tissue injuries and stimulates the production of acute phase proteins such as C-reactive protein (CRP), which is commonly used as a clinical marker of inflammation. Elevated IL-6 levels are associated with an increased risk of myocardial infarction, heart failure, and overall mortality in CVD patients. IL-6 not only reflects inflammatory status but also contributes to the progression of atherosclerosis by promoting endothelial dysfunction and plaque instability. IL-1β, another key pro-inflammatory cytokine, is primarily produced by activated macrophages. It promotes endothelial cell activation and leukocyte recruitment, thereby accelerating the atherosclerotic process. Elevated IL-1β levels are associated with adverse outcomes in coronary artery disease and heart failure, highlighting its role in both the initiation and progression of atherosclerosis. The inhibition of IL-1β has shown promise in reducing recurrent cardiovascular events. IL-6 and IL-1β influence various stages of atherosclerosis, from the early recruitment of leukocytes to the development of advanced atherosclerotic lesions. IL-6, through its receptor IL-6R, can activate several downstream signaling pathways, including the JAK/STAT pathway, which leads to the expression of genes involved in inflammation and cell survival. Chronic IL-6 signaling contributes to the maintenance of a pro-inflammatory environment, which exacerbates endothelial dysfunction and promotes the development of unstable atherosclerotic plaques. IL-1β, on the other hand, is a crucial mediator of the inflammasome pathway. Upon activation by danger signals such as cholesterol crystals, the NLRP3 inflammasome assembles and processes pro-IL-1β into its active form. IL-1β then acts on endothelial cells to increase the expression of adhesion molecules, promoting the recruitment of monocytes and their differentiation into macrophages. These macrophages ingest oxidized low-density lipoproteins (oxLDL) and transform into foam cells, a hallmark of early atherosclerotic lesions. Moreover, IL-1β enhances the production of other pro-inflammatory cytokines, amplifying the inflammatory response and further driving atherosclerosis. In summary, both IL-6 and IL-1β are integral to the inflammatory processes that underlie cardiovascular diseases.

Recent clinical trials have demonstrated the efficacy of certain well-known anti-inflammatory drugs in improving cardiovascular outcomes. The CANTOS trial, for example, showed that canakinumab, an IL-1β inhibitor, significantly reduced recurrent cardiovascular events in patients with a history of myocardial infarction. This trial highlighted the potential of targeting IL-1β as a therapeutic strategy in cardiovascular disease management. Additionally, the Colchicine Cardiovascular Outcomes Trial (COLCOT) revealed that low-dose colchicine, an anti-inflammatory drug commonly used to treat gout, effectively reduced the risk of recurrent ischemic cardiovascular events in patients with a recent myocardial infarction. This finding suggests that colchicine could be repurposed as a beneficial therapy for cardiovascular conditions.

Furthermore, the IL-6 receptor antagonist tocilizumab has been investigated in the ASSAIL-MI trial, which aimed to assess its effects on patients with ST-elevation myocardial infarction (STEMI). The trial found that early administration of tocilizumab improved myocardial salvage and reduced systemic inflammation, as indicated by lower levels of high-sensitivity C-reactive protein (hsCRP).

These studies underscore the importance of anti-inflammatory therapies in managing cardiovascular diseases and highlight the need for further research to optimize treatment strategies. Personalized medicine approaches, which tailor anti-inflammatory treatments based on individual patient profiles, including their inflammatory status, could further enhance the effectiveness of these interventions [2,26,41,44,45].

Recent studies have highlighted the importance of a personalized approach to patient care, particularly in the context of acute myocardial infarction (AMI). Myocardial inflammation is now recognized as a prognostic and therapeutic target in AMI. For instance, research has shown that myocardial inflammation, as measured by specific biomarkers, can predict outcomes in patients with AMI. Targeting inflammation in these patients has been proposed as a strategy to improve prognosis and reduce the risk of recurrent cardiovascular events.

Personalized treatment strategies that tailor anti-inflammatory therapies based on individual patient profiles, including their inflammatory status and specific biomarker levels, could enhance the effectiveness of interventions and improve clinical outcomes. This approach aligns with the broader trend in medicine towards precision health, where treatments are customized to the unique characteristics of each patient, leading to more effective and targeted therapies.

Recent studies have highlighted the importance of a personalized approach to patient care, particularly in the context of acute myocardial infarction (AMI). Myocardial inflammation is now recognized as a prognostic and therapeutic target in AMI. For instance, research has shown that myocardial inflammation, as measured by specific biomarkers, can predict outcomes in patients with AMI. Targeting inflammation in these patients has been proposed as a strategy to improve prognosis and reduce the risk of recurrent cardiovascular events. In the context of AMI, inflammation can be both beneficial and harmful. The initial inflammatory response helps to clear necrotic tissue and initiate repair, but excessive inflammation can lead to adverse outcomes, including worse myocardial remodeling and increased risk of heart failure. Recent studies, including those by Matter et al. (2023), emphasize that a balanced approach is necessary to manage inflammation in AMI patients. Matter et al. (2023) describe how early anti-inflammatory interventions targeting IL-1 and IL-6 pathways have shown promise in improving outcomes by reducing excessive inflammation. For example, IL-1 and IL-6 inhibitors can help mitigate the harmful effects of an excessive inflammatory response immediately following AMI. The study also highlights that personalized treatment strategies, which consider the patient’s inflammatory burden and the timing of intervention, are crucial for optimizing therapeutic outcomes. By identifying patients with a high pro-inflammatory burden and initiating treatment early (within 12 h post-AMI), it is possible to improve patient outcomes significantly. These insights suggest that incorporating anti-inflammatory therapies as part of a personalized treatment plan for AMI patients could enhance the standard care protocols. Further research and clinical trials are necessary to determine the most effective treatment regimens and to validate these findings in larger patient populations [46,47].

Our review included 12 studies that met the inclusion criteria, providing robust evidence on the association between IL-6 and IL-1β levels and cardiovascular outcomes. Elevated IL-6 levels were strongly linked to peripheral artery disease (PAD), myocardial infarction, and heart failure. Studies such as those by Signorelli et al. (2016) and Takamura et al. (2017) demonstrated significant elevations in IL-6 levels in PAD patients and post-endovascular therapy, respectively, indicating that IL-6 is a key marker of inflammation in these conditions. Similarly, IL-1β was found to be elevated in patients with coronary artery disease and heart failure, with studies by Gotsman et al. (2014) and Ridker et al. (2017) showing that IL-1β levels are predictive of worse outcomes in these populations [20,29].

The meta-analysis conducted in this review further supports the role of IL-6 as a critical biomarker for cardiovascular disease progression. The summary effect size indicated a significant association between higher IL-6 levels and increased risk of adverse cardiovascular events. This finding aligns with previous research that has identified IL-6 as a central player in the inflammatory response associated with atherosclerosis and other cardiovascular conditions. The clinical implications of these findings are profound. Anti-inflammatory therapies targeting IL-6 and IL-1β pathways hold promise for reducing the inflammatory burden and improving cardiovascular outcomes. The CANTOS trial, which demonstrated that canakinumab (an IL-1β inhibitor) significantly reduced recurrent cardiovascular events in patients with a history of myocardial infarction, highlights the potential benefits of such therapeutic approaches. Similar interventions targeting IL-6 are being explored, and early results are promising. Given the complexity of inflammatory pathways and the multifaceted roles of IL-6 and IL-1β, it is crucial to identify specific patient populations that may benefit the most from these therapies. [23,48,49].

Personalized approaches to patient care, especially in the context of acute myocardial infarction (AMI), are gaining recognition. Myocardial inflammation is now considered a prognostic and therapeutic target in AMI. Recent studies have shown that myocardial inflammation, measured by specific biomarkers, can predict outcomes in AMI patients. For instance, Matter et al. (2023) highlight the significance of targeting myocardial inflammation to improve patient outcomes. Their research demonstrates that anti-inflammatory treatments, particularly those targeting IL-1 and IL-6 pathways, can reduce the adverse effects of excessive inflammation following AMI. Implementing a personalized approach, where treatments are tailored based on the patient’s inflammatory profile and initiated early (within 12 h post-AMI), has shown promising results in reducing recurrent cardiovascular events and improving prognosis [6,7,50].

This systematic review has several strengths, including a comprehensive literature search, rigorous inclusion and exclusion criteria, and a detailed quality assessment of included studies. The use of a meta-analysis to quantify the association between IL-6 levels and cardiovascular outcomes adds robustness to the findings. However, there are also limitations to consider. The heterogeneity among studies, including differences in study design, population characteristics, and methods for measuring cytokine levels, may affect the generalizability of the results. Additionally, the observational nature of most included studies precludes establishing causality. Further randomized controlled trials are needed to confirm the benefits of targeting IL-6 and IL-1β in reducing cardiovascular events.

Despite the significant findings of our review, several limitations should be acknowledged. The included studies varied in their design, population size, and methodologies for measuring cytokine levels, leading to potential heterogeneity in the results. Additionally, most studies were observational, which precludes establishing a direct causal relationship between elevated cytokine levels and cardiovascular outcomes. The varying follow-up durations and differences in baseline characteristics of study populations further complicate the generalizability of the findings. Future research should focus on conducting large-scale, randomized controlled trials to validate these associations and to determine the causal effects of cytokine modulation on cardiovascular outcomes.

Future research should focus on further elucidating the mechanistic pathways through which IL-6 and IL-1β contribute to cardiovascular disease progression. This includes exploring the interactions between these cytokines and other inflammatory mediators, as well as their effects on different stages of atherosclerosis and heart failure. Additionally, clinical trials investigating the efficacy of IL-6 and IL-1β inhibitors in diverse patient populations are essential to establish their therapeutic potential and identify optimal treatment strategies. Investigating the potential of other inflammatory markers, such as TNF-α, MCP-1, and CRP, could provide a more comprehensive understanding of the inflammatory landscape in cardiovascular diseases. Personalized medicine approaches, which tailor treatments based on individual inflammatory profiles, should be further developed and tested in clinical trials. These strategies have the potential to improve the efficacy of anti-inflammatory therapies and to reduce the risk of adverse cardiovascular events.

**Figure 3:**
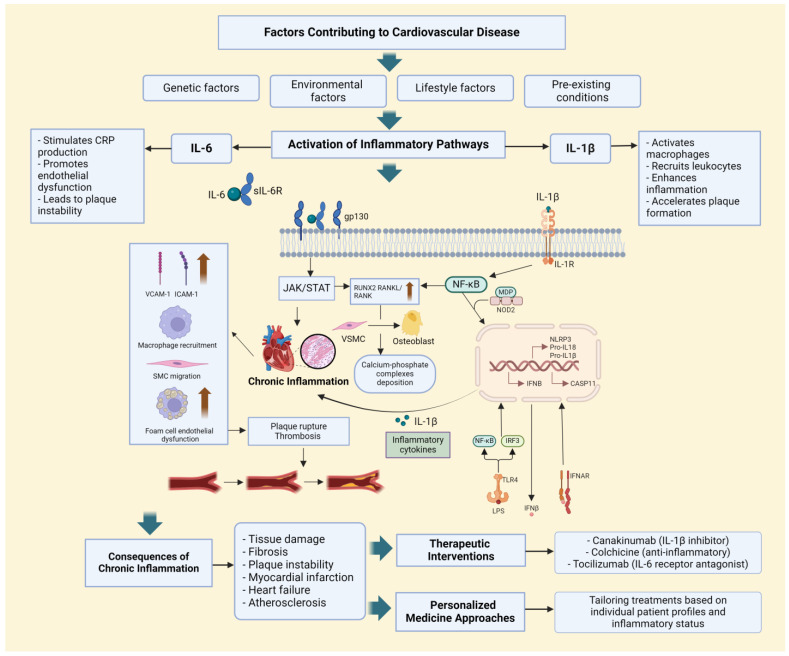
Mechanisms of IL-6 and IL-1β in cardiovascular disease.

Apart from IL-6 and IL-1β, other inflammatory markers may also play critical roles in cardiovascular diseases. Tumor necrosis factor-alpha (TNF-α) is another key cytokine involved in systemic inflammation and has been implicated in the pathogenesis of heart failure and atherosclerosis. Monocyte chemoattractant protein-1 (MCP-1) is crucial for recruiting monocytes to sites of inflammation and has been associated with the development of atherosclerotic plaques. High-sensitivity C-reactive protein (hsCRP) is widely used as a clinical marker of inflammation and has been shown to predict cardiovascular events (Figure 3). Exploring the roles of these and other inflammatory markers could provide new insights into the mechanisms of cardiovascular diseases and identify additional therapeutic targets [8,51,52].

Factors contributing to cardiovascular disease include genetic factors, environmental factors, lifestyle factors, and pre-existing conditions. These factors lead to the activation of inflammatory pathways. IL-6 stimulates CRP production, promotes endothelial dysfunction, and leads to plaque instability. IL-6 binds to its soluble receptor (sIL-6R), forming a complex that interacts with gp130 on the cell surface, activating the JAK/STAT signaling cascade. This results in chronic inflammation, endothelial dysfunction, macrophage recruitment, and smooth muscle cell (SMC) migration, which contribute to foam cell formation and plaque instability. IL-1β activates macrophages, recruits leukocytes, enhances inflammation, and accelerates plaque formation. IL-1β binds to the IL-1 receptor (IL-1R) on the cell surface, activating the NF-κB signaling pathway, which leads to the activation of the NLRP3 inflammasome and converts pro-IL-1β into its active form, further enhancing the inflammatory response. The figure also highlights the role of RUNX2, RANKL/RANK, and osteoblasts in the deposition of calcium-phosphate complexes and vascular calcification. Lipopolysaccharides (LPSs) from bacterial infections can enhance the inflammatory response by stimulating both IL-6 and IL-1β pathways, exacerbating cardiovascular inflammation and disease progression through increased cytokine production and systemic inflammation. Interferon-β (IFN-β) interacts with its receptor IFNAR, activating downstream signaling pathways that modulate inflammation and immune responses, thereby playing a role in regulating inflammatory processes in cardiovascular diseases. Therapeutic interventions targeting IL-6 and IL-1β include canakinumab (an IL-1β inhibitor), colchicine (an anti-inflammatory drug), and tocilizumab (an IL-6 receptor antagonist). These treatments aim to reduce inflammation and improve cardiovascular outcomes. The figure emphasizes the importance of personalized medicine approaches, where treatments are tailored based on individual patient profiles and inflammatory status, enhancing the efficacy of anti-inflammatory therapies and reducing the risk of adverse cardiovascular events [53,54].

## 5. Conclusions

In conclusion, this systematic review provides compelling evidence that IL-6 and IL-1β are key inflammatory mediators associated with adverse cardiovascular outcomes. Elevated levels of these cytokines are linked to increased risks of myocardial infarction, heart failure, and overall mortality. Targeting these cytokines offers a promising approach to reducing the inflammatory burden and improving clinical outcomes in patients with cardiovascular diseases. Continued research and clinical trials are essential to translate these findings into effective therapeutic strategies and enhance the management of cardiovascular diseases. Further research is needed to elucidate the precise mechanisms by which IL-6 and IL-1β contribute to CVD progression, and clinical trials should be conducted to evaluate the efficacy of IL-6 and IL-1β inhibitors in diverse patient populations. Personalized medicine approaches should be developed to tailor anti-inflammatory therapies based on individual patient profiles, and healthcare providers should consider the potential benefits of targeting IL-6 and IL-1β in patients with a high inflammatory burden to improve cardiovascular outcomes.

## Figures and Tables

**Table 1:** Characteristics of included studies and their key findings.

Author and Year	Study Design	Number of Participants	Age of Participants	Key Findings	IL-6 Level	IL-1β Level	Conclusions
Djahanpour, 2023 [19]	Systematic review	17 studies	Various	IL-6 and IL-8 were most strongly associated with PAD	Elevated	-	IL-6 and IL-8 may serve as biomarkers for PAD diagnosis and prognosis
Signorelli, 2016 [20]	Cohort study	80	Mean age 68	Significant elevation in IL-6 and other inflammatory markers in PAD patients	Elevated	-	High levels of IL-6 and other inflammatory markers associated with PAD
Takamura, 2017 [21]	Cohort study	35	Mean age 65	Significant elevation in IL-6, hs-CRP, and D-dimer post-endovascular therapy	Elevated 36 h post-therapy	-	IL-6 may be a marker for predicting outcomes post-endovascular therapy
Gremmels, 2019 [22]	Cohort study	118 (PAD) and 108 (CLTI)	Mean age 70	IL-6 and IL-8 associated with increased risk of amputation	Elevated	-	IL-6 and IL-8 may be prognostic markers for assessing amputation risk in CLTI patients
Martínez, 2015 [23]	Randomized controlled trial	40 (ACS), 33 (Stable CAD), 10 (Controls)	Mean age 58	Colchicine significantly reduced IL-6, IL-1β, and IL-18 levels in ACS patients	Reduced	Reduced	Colchicine rapidly reduces inflammatory cytokines in ACS patients
Gotsman, 2014 [24]	Cohort study	101	Mean age 65	Elevated IL-6 and IL-1β levels were associated with worse outcomes in heart failure patients	Elevated	Elevated	High IL-6 and IL-1β levels are linked to poor prognosis in heart failure
Sokolik 2021 [15]	Cohort study	150	Various	Impact of IL-6 genetic variants on methotrexate treatment efficacy in psoriatic arthritis	Elevated	-	L-6 genetic variants influence treatment efficacy and cardiovascular risk management
Guo, 2020 [25]	Cohort study	68	Mean age 67	Elevated IL-6 levels were better predictors of in-stent restenosis than hs-CRP	Elevated	-	IL-6 may be a better predictor of in-stent restenosis compared to hs-CRP
Tardif, 2019 [26]	Randomized controlled trial	2497	Mean age 60	Colchicine reduced IL-6 levels in post-myocardial infarction patients	Reduced	-	Colchicine may reduce inflammation by lowering IL-6 levels post-MI
Jones, 2023 [27]	Cohort study	50 (PAD patients)	Mean age 63	Elevated IL-6 levels in PAD patients, associated with increased risk of cardiovascular events	Elevated	-	Elevated IL-6 levels are significantly associated with inflammation and higher risk of cardiovascular events in PAD patients
DePalma, 2021 [28]	Cohort study	100	Mean age 66	Ferritin levels coincided with IL-6 levels in PAD patients	Elevated	-	High ferritin and IL-6 levels are linked with inflammation in PAD
Ridker, 2017 [29]	Randomized controlled trial	10061	Mean age 61	Canakinumab reduced IL-6 and IL-1β levels, leading to lower cardiovascular event rates.	Reduced	Reduced	IL-1β inhibition reduces cardiovascular events by lowering IL-6 and IL-1β levels.

## Data Availability

Data supporting the reported results are available from the corresponding author upon request.

## References

[1] Haller P.M., Beer B.N., Tonkin A.M., Blankenberg S., Neumann J.T. (2021). Role of Cardiac Biomarkers in Epidemiology and Risk Outcomes. Clin. Chem..

[2] Ait-Oufella H., Libby P., Tedgui A. (2019). Anticytokine Immune Therapy and Atherothrombotic Cardiovascular Risk. Arterioscler. Thromb. Vasc. Biol..

[3] Parish E., Bloom T., Godlee F. (2015). Statins for people at low risk. BMJ.

[4] Blais J.E., Chan E.W., Law S.W.Y., Mok M.T., Huang D., Wong I.C.K., Siu C.W. (2019). Trends in statin prescription prevalence, initiation, and dosing: Hong Kong, 2004–2015. Atherosclerosis.

[5] Salami J.A., Warraich H.J., Valero-Elizondo J., Spatz E.S., Desai N.R., Rana J.S., Virani S.S., Blankstein R., Khera A., Blaha M.J. (2018). National Trends in Nonstatin Use and Expenditures Among the US Adult Population From 2002 to 2013: Insights From Medical Expenditure Panel Survey. J. Am. Heart Assoc..

[6] Hansson G.K. (2005). Inflammation, atherosclerosis, and coronary artery disease. N. Engl. J. Med..

[7] Blankenberg S., Zeller T., Saarela O., Havulinna A.S., Kee F., Tunstall-Pedoe H., Kuulasmaa K., Yarnell J., Schnabel R.B., Wild P.S. (2010). Contribution of 30 biomarkers to 10-year cardiovascular risk estimation in 2 population cohorts: The MONICA, risk, genetics, archiving, and monograph (MORGAM) biomarker project. Circulation.

[8] Ridker P.M., Buring J.E., Rifai N., Cook N.R. (2007). Development and validation of improved algorithms for the assessment of global cardiovascular risk in women: The Reynolds Risk Score. JAMA.

[9] Tabrizi R., Tamtaji O.R., Mirhosseini N., Lankarani K.B., Akbari M., Dadgostar E., Borhani-Haghighi A., Peymani P., Ahmadizar F., Asemi Z. (2019). The effects of statin use on inflammatory markers among patients with metabolic syndrome and related disorders: A systematic review and meta-analysis of randomized controlled trials. Pharmacol. Res..

[10] Zhang L., Zhang S., Jiang H., Sun A., Wang Y., Zou Y., Ge J., Chen H. (2010). Effects of statin therapy on inflammatory markers in chronic heart failure: A meta-analysis of randomized controlled trials. Arch. Med. Res..

[11] Milajerdi A., Larijani B., Esmaillzadeh A. (2019). Statins influence biomarkers of low grade inflammation in apparently healthy people or patients with chronic diseases: A systematic review and meta-analysis of randomized clinical trials. Cytokine.

[12] Libby P. (2002). Inflammation in atherosclerosis. Nature.

[13] Bartolomaeus H., Avery E.G., Bartolomaeus T.U.P., Kozhakhmetov S., Zhumadilov Z., Müller D.N., Wilck N., Kushugulova A., Forslund S.K. (2020). Blood pressure changes correlate with short-chain fatty acid production potential shifts under a synbiotic intervention. Cardiovasc. Res..

[14] Curtis P.J., Kroon P.A., Hollands W.J., Walls R., Jenkins G., Kay C.D., Cassidy A. (2009). Cardiovascular disease risk biomarkers and liver and kidney function are not altered in postmenopausal women after ingesting an elderberry extract rich in anthocyanins for 12 weeks. J. Nutr..

[15] Sokolik R., Iwaszko M., Świerkot J., Wysoczańska B., Korman L., Wiland P., Bogunia-Kubik K. (2021). Relationship Between Interleukin-6-174G/C Genetic Variant and Efficacy of Methotrexate Treatment in Psoriatic Arthritis Patients. Pharmgenomics Pers. Med..

[16] Wan X., Wang W., Liu J., Tong T. (2014). Estimating the sample mean and standard deviation from the sample size, median, range and/or interquartile range. BMC Med. Res. Methodol..

[17] Willeit P., Welsh P., Evans J.D.W., Tschiderer L., Boachie C., Jukema J.W., Ford I., Trompet S., Stott D.J., Kearney P.M. (2017). High-Sensitivity Cardiac Troponin Concentration and Risk of First-Ever Cardiovascular Outcomes in 154,052 Participants. J. Am. Coll. Cardiol..

[18] Page M.J., McKenzie J.E., Bossuyt P.M., Boutron I., Hoffmann T.C., Mulrow C.D., Shamseer L., Tetzlaff J.M., Akl E.A., Brennan S.E. (2021). The PRISMA 2020 statement: An updated guideline for reporting systematic reviews. BMJ.

[19] Djahanpour N., Ahsan N., Li B., Khan H., Connelly K., Leong-Poi H., Qadura M. (2023). A Systematic Review of Interleukins as Diagnostic and Prognostic Biomarkers for Peripheral Artery Disease. Biomolecules.

[20] Signorelli S.S., Anzaldi M., Libra M., Navolanic P.M., Malaponte G., Mangano K., Quattrocchi C., Di Marco R., Fiore V., Neri S. (2016). Plasma Levels of Inflammatory Biomarkers in Peripheral Arterial Disease: Results of a Cohort Study. Angiology.

[21] Takamura T.A., Tsuchiya T., Oda M., Watanabe M., Saito R., Sato-Ishida R., Akao H., Kawai Y., Kitayama M., Kajinami K. (2017). Circulating malondialdehyde-modified low-density lipoprotein (MDA-LDL) as a novel predictor of clinical outcome after endovascular therapy in patients with peripheral artery disease (PAD). Atherosclerosis.

[22] Gremmels H., Teraa M., de Jager S.C.A., Pasterkamp G., de Borst G.J., Verhaar M.C. (2019). A Pro-Inflammatory Biomarker-Profile Predicts Amputation-Free Survival in Patients with Severe Limb Ischemia. Sci. Rep..

[23] Martínez G.J., Robertson S., Barraclough J., Xia Q., Mallat Z., Bursill C., Celermajer D.S., Patel S. (2015). Colchicine Acutely Suppresses Local Cardiac Production of Inflammatory Cytokines in Patients With an Acute Coronary Syndrome. J. Am. Heart Assoc..

[24] Gotsman I., Shauer A., Lotan C., Keren A. (2014). Impaired fasting glucose: A predictor of reduced survival in patients with heart failure. Eur. J. Heart Fail..

[25] Guo S., Zhang Z., Wang L., Yuan L., Bao J., Zhou J., Jing Z. (2020). Six-month results of stenting of the femoropopliteal artery and predictive value of interleukin-6: Comparison with high-sensitivity C-reactive protein. Vascular.

[26] Tardif J.C., Kouz S., Waters D.D., Bertrand O.F., Diaz R., Maggioni A.P., Pinto F.J., Ibrahim R., Gamra H., Kiwan G.S. (2019). Efficacy and Safety of Low-Dose Colchicine after Myocardial Infarction. N. Engl. J. Med..

[27] Parry-Jones A.R., Stocking K., MacLeod M.J., Clarke B., Werring D.J., Muir K.W., Vail A. (2023). Phase II randomised, placebo-controlled, clinical trial of interleukin-1 receptor antagonist in intracerebral haemorrhage: BLOcking the Cytokine IL-1 in ICH (BLOC-ICH). Eur. Stroke J..

[28] DePalma R.G., Hayes V.W., O’Leary T.J. (2021). Optimal serum ferritin level range: Iron status measure and inflammatory biomarker. Metallomics.

[29] Ridker P.M., Everett B.M., Thuren T., MacFadyen J.G., Chang W.H., Ballantyne C., Fonseca F., Nicolau J., Koenig W., Anker S.D. (2017). Antiinflammatory Therapy with Canakinumab for Atherosclerotic Disease. N. Engl. J. Med..

[30] Libra M., Signorelli S.S., Bevelacqua Y., Navolanic P.M., Bevelacqua V., Polesel J., Talamini R., Stivala F., Mazzarino M.C., Malaponte G. (2006). Analysis of G(-174)C IL-6 polymorphism and plasma concentrations of inflammatory markers in patients with type 2 diabetes and peripheral arterial disease. J. Clin. Pathol..

[31] Ridker P.M., Paynter N.P., Rifai N., Gaziano J.M., Cook N.R. (2008). C-reactive protein and parental history improve global cardiovascular risk prediction: The Reynolds Risk Score for men. Circulation.

[32] Goldsborough E., Osuji N., Blaha M.J. (2022). Assessment of Cardiovascular Disease Risk: A 2022 Update. Endocrinol. Metab. Clin. N. Am..

[33] Soysal P., Arik F., Smith L., Jackson S.E., Isik A.T. (2020). Inflammation, Frailty and Cardiovascular Disease. Adv. Exp. Med. Biol..

[34] Ramallal R., Toledo E., Martínez-González M.A., Hernández-Hernández A., García-Arellano A., Shivappa N., Hébert J.R., Ruiz-Canela M. (2015). Dietary Inflammatory Index and Incidence of Cardiovascular Disease in the SUN Cohort. PLoS ONE.

[35] Van Taunay J.S., Albelda M.T., Frias J.C., Lipinski M.J. (2018). Biologics and Cardiovascular Disease. J. Cardiovasc. Pharmacol..

[36] Haybar H., Bandar B., Torfi E., Mohebbi A., Saki N. (2023). Cytokines and their role in cardiovascular diseases. Cytokine.

[37] Apostolakis S., Vogiatzi K., Krambovitis E., Spandidos D.A. (2008). IL-1 cytokines in cardiovascular disease: Diagnostic, prognostic and therapeutic implications. Cardiovasc. Hematol. Agents Med. Chem..

[38] Pfeiler S., Winkels H., Kelm M., Gerdes N. (2019). IL-1 family cytokines in cardiovascular disease. Cytokine.

[39] Caiazzo E., Sharma M., Rezig A.O.M., Morsy M.I., Czesnikiewicz-Guzik M., Ialenti A., Sulicka-Grodzicka J., Pellicori P., Crouch S.H., Schutte A.E. (2024). Circulating cytokines and risk of developing hypertension: A systematic review and meta-analysis. Pharmacol. Res..

[40] Ridker P.M. (2020). Targeting Interleukin-1 and Interleukin-6: The Time Has Come to Aggressively Address Residual Inflammatory Risk. J. Am. Coll. Cardiol..

[41] Pearson T.A., Mensah G.A., Alexander R.W., Anderson J.L., Cannon R.O., Criqui M., Fadl Y.Y., Fortmann S.P., Hong Y., Myers G.L. (2003). Markers of inflammation and cardiovascular disease: Application to clinical and public health practice: A statement for healthcare professionals from the Centers for Disease Control and Prevention and the American Heart Association. Circulation.

[42] Kreiner F.F., Kraaijenhof J.M., von Herrath M., Hovingh G.K.K., von Scholten B.J. (2022). Interleukin 6 in diabetes, chronic kidney disease, and cardiovascular disease: Mechanisms and therapeutic perspectives. Expert. Rev. Clin. Immunol..

[43] Popkova T.V., Novikova D.S., Nasonov E.L. (2016). Interleukin-6 inhibition and cardiovascular disease in patients with rheumatoid arthritis. Ter. Arkh..

[44] Samuel M., Tardif J.C. (2021). Lessons learned from large Cardiovascular Outcome Trials targeting inflammation in cardiovascular disease (CANTOS, CIRT, COLCOT and LoDoCo2). Futur. Cardiol..

[45] Yang T.C., Chang P.Y., Lu S.C. (2017). L5-LDL from ST-elevation myocardial infarction patients induces IL-1β production via LOX-1 and NLRP3 inflammasome activation in macrophages. Am. J. Physiol. Heart Circ. Physiol..

[46] Bergamaschi L., Landi A., Maurizi N., Pizzi C., Leo L.A., Arangalage D., Iglesias J.F., Eeckhout E., Schwitter J., Valgimigli M. (2024). Acute Response of the Noninfarcted Myocardium and Surrounding Tissue Assessed by T2 Mapping After STEMI. JACC Cardiovasc. Imaging.

[47] Matter M.A., Paneni F., Libby P., Frantz S., Stähli B.E., Templin C., Mengozzi A., Wang Y.J., Kündig T.M., Räber L. (2024). Inflammation in acute myocardial infarction: The good, the bad and the ugly. Eur. Heart J..

[48] Wytrykowska A., Prosba-Mackiewicz M., Nyka W.M. (2016). IL-1β, TNF-α, and IL-6 levels in gingival fluid and serum of patients with ischemic stroke. J. Oral Sci..

[49] Nong Y., Wei X., Yu D. (2023). Inflammatory mechanisms and intervention strategies for sepsis-induced myocardial dysfunction. Immun. Inflamm. Dis..

[50] Shetty G.K., Economides P.A., Horton E.S., Mantzoros C.S., Veves A. (2004). Circulating adiponectin and resistin levels in relation to metabolic factors, inflammatory markers, and vascular reactivity in diabetic patients and subjects at risk for diabetes. Diabetes Care.

[51] Layden J., Michaels J., Bermingham S., Higgins B. (2012). Diagnosis and management of lower limb peripheral arterial disease: Summary of NICE guidance. BMJ.

[52] Ridker P.M., Devalaraja M., Baeres F.M.M., Engelmann M.D.M., Hovingh G.K., Ivkovic M., Lo L., Kling D., Pergola P., Raj D. (2021). IL-6 inhibition with ziltivekimab in patients at high atherosclerotic risk (RESCUE): A double-blind, randomised, placebo-controlled, phase 2 trial. Lancet.

[53] Mishra S., Kass D.A. (2021). Cellular and molecular pathobiology of heart failure with preserved ejection fraction. Nat. Rev. Cardiol..

[54] Feng Y., Ye D., Wang Z., Pan H., Lu X., Wang M., Xu Y., Yu J., Zhang J., Zhao M. (2022). The Role of Interleukin-6 Family Members in Cardiovascular Diseases. Front. Cardiovasc. Med..

